# Enantiomeric Isoflavones with neuroprotective activities from the Fruits of *Maclura tricuspidata*

**DOI:** 10.1038/s41598-018-36095-8

**Published:** 2019-02-11

**Authors:** Nguyen Tuan Hiep, Jaeyoung Kwon, Sungeun Hong, Nahyun Kim, Yuanqiang Guo, Bang Yeon Hwang, Woongchon Mar, Dongho Lee

**Affiliations:** 1 Department of Biosystems and Biotechnology, College of Life Science and Biotechnology, Korea University, Seoul, 02841 Republic of Korea; 2 Department of Extraction Technology, Vietnam National Institute of Medicinal Materials, 3B Quang Trung, Hoan Kiem, Hanoi Vietnam; 3 Natural Constituents Research Center, Korea Institute of Science and Technology (KIST), Gangneung, 25451 Republic of Korea; 4 Natural Products Research Institute, College of Pharmacy, Seoul National University, Seoul, 151-742 Republic of Korea; 5 Forest Medicinal Resources Research Center, National Institute of Forest Science, Yeongju, 36040 Republic of Korea; 6 State Key Laboratory of Medicinal Chemical Biology, College of Pharmacy, and Tianjin Key Laboratory of Molecular Drug Research, Nankai University, Tianjin, 300350 People’s Republic of China; 7 College of Pharmacy, Chungbuk National University, Cheongju, 361-763 Republic of Korea

## Abstract

Seven pairs of enantiomeric isoflavones (1a/1b–7a/7b) were obtained from the ethyl acetate extract of the fruits of *Maclura tricuspidata* (syn. *Cudrania tricuspidata*), and successfully separated by chiral high-pressure liquid chromatography (HPLC). The structures and absolute configurations of the enantiomeric isoflavones were established on the basic of comprehensive spectroscopic analyses and quantum chemical calculation methods. Compounds 1, 1a, and 1b exhibited neuroprotective activities against oxygen-glucose deprivation/reoxygenation (ODG/R)-induced SH-SY5Y cells death with EC_50_ values of 5.5 µM, 4.0 µM, and 10.0 µM, respectively. Furthermore, 1, 1a, and 1b inhibited OGD/R-induced reactive oxygen species generation in SH-5Y5Y cells with IC_50_ values of 6.9 µM, 4.5 µM, and 9.5 µM, respectively.

## Introduction

*Maclura tricuspidata* (Carr.) Bur. (syn. *Cudrania tricuspidata*) is a perennial plant, which is mainly distributed in the southern part of Korea. It has been used as folk remedies for gastritis, liver damage, and hypertension in Korean traditional medicine^1^. Currently, its fruits are consumed fresh and in juices and jams. Further development as a dietary supplement and functional food ingredient has been actively accomplished in many fields^2^. According to previous reports, various types of flavonoids, including isoflavones^3–7^, along with xanthones^8–12^ are considered as the major bioactive constituents of *M. tricuspidata*, exhibiting antioxidant^8^, antitherosclerotic, anti-inflammatory^13^, cytotoxic^10^, hepatoprotective^14^, and neuroprotective activities^6,7,11,12^.

Cerebral ischemia, also known as brain ischemia or ischemic stroke, is one of the most common causes of mortality and morbidity, conducing to major negative social and economic consequences. Accordingly, the prevention of this disease is clearly an important public health priority. It occurs as a result of the cerebral blood flow is disrupted, leading to the starvation of oxygen and glucose to the affected area, causing of irreversible brain damage^15,16^. Thus far, knowledge about the mechanisms of ischemic brain damage has increased considerably. In general, during ischemia a variety of pathophysiological mechanisms such as calcium influx, glutamate excitotoxicity, inflammation, mitochondrial dysfunction, and oxidative stress were activated, leading to neuronal cell death^17–19^.

In present study, seven pairs of enantiomeric isoflavones (**1a/1b–7a/7b**) were obtained from the ethyl acetate extract of the fruits of *M. tricuspidata*. These enantiomeric isoflavones were further purified by using chiral high-pressure liquid chromatography (HPLC), their structures with absolute configurations were established based on interpretation of their 1D and 2D NMR, and HRESIMS data together with electronic circular dichroism (ECD) calculations. Furthermore, the neuroprotective potentials of the isolated compounds were evaluated.

## Results and Discussion

**Figure 1:**
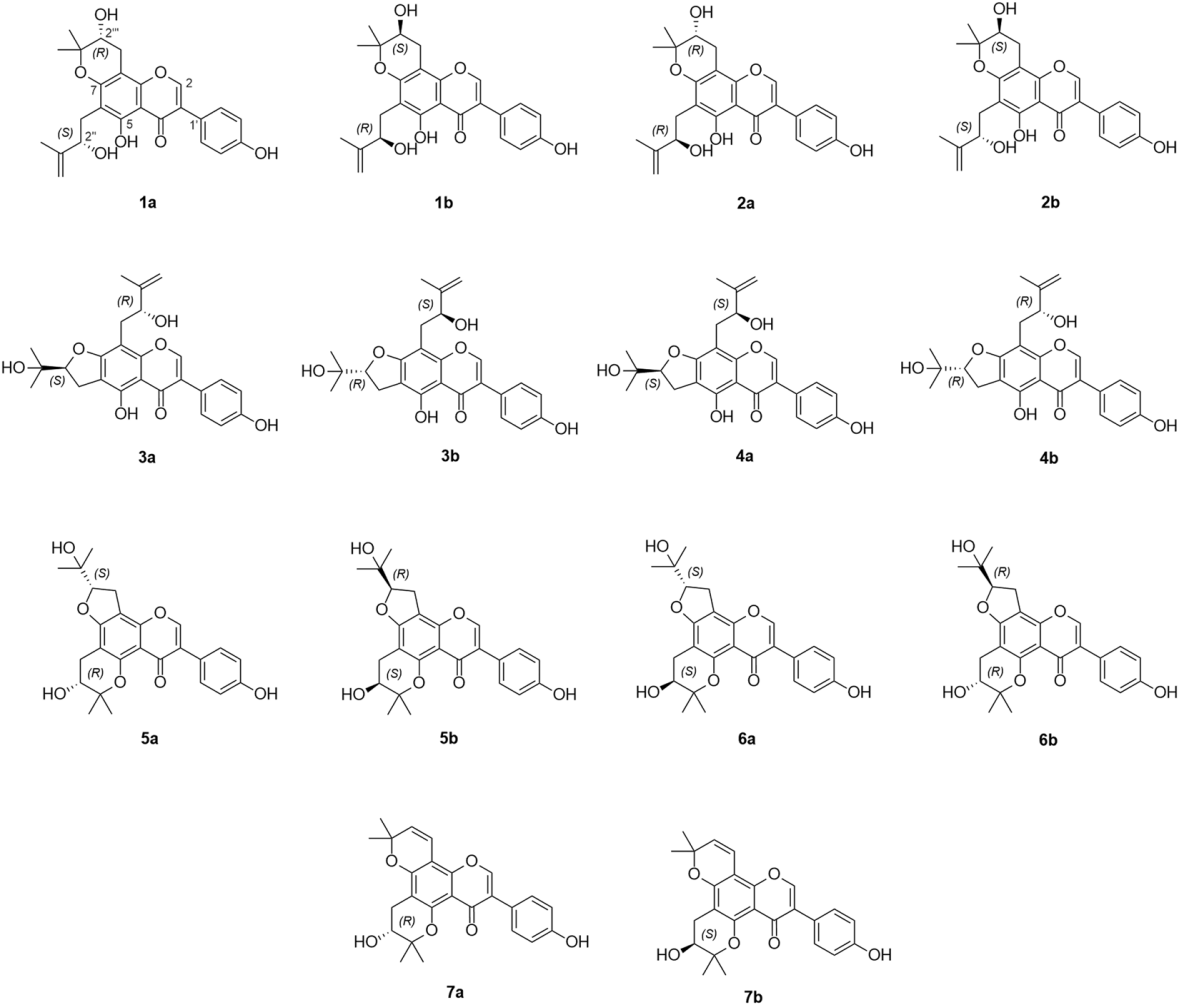
Structures of enantiomeric isoflavones **1a–7b**.

Compound **1** was determined as C_25_H_26_O_7_ by the HRESIMS [M + H]^+^ ion at *m/z* 439.1742 (calcd. for C_25_H_25_O_7_, 439.1757). The ^1^H and ^13^C NMR spectra resembled those of cudraisoflavone D (Supplementary S.24, Table 1)^6^, except for the appearance of a 3-hydroxy-2,2-dimethyldihydropyran group [*δ*_H_ 3.07 (1 H, dd, *J* = 16.5, 5.5 Hz, Ha-1′′′), 2.73 (1 H, dd, *J* = 16.5, 7.5 Hz, Hb-1′′′), 3.89 (1 H, dd, *J* = 7.5, 5.0 Hz, H-2′′′), 1.35 (3 H, s, Me-4′′′), and 1.45 (3 H, s, Me-5′′′)] at the C-7 and C-8 positions instead of the furan group, as deduced from the HMBC correlations H-1′′′/C-7 (*δ*_C_ 158.3), C-8 (*δ*_C_ 99.2), and C-9 (*δ*_C_ 154.5). Based on these, compound **1** was established as depicted (Fig. 1) and named cudraisoflavone U.

**Table 1:** ^1^H and ^13^C NMR spectroscopic data of compounds **1–7**.

No.	1 (Acetone-*d*_6_)		2 (Acetone-*d*_6_)		3 (Acetone-*d*_6_)		4 (Acetone-*d*_6_)		5 (DMSO-*d*_6_)		6 (DMSO-*d*_6_)		7 (DMSO-*d*_6_)	
	*δ*_C_	*δ*_H_ (*J* in Hz)	*δ*_C_	*δ*_H_ (*J* in Hz)	*δ*_C_	*δ*_H_ (*J* in Hz)	*δ*_C_	*δ*_H_ (*J* in Hz)	*δ*_C_	*δ*_H_ (*J* in Hz)	*δ*_C_	*δ*_H_ (*J* in Hz)	*δ*_C_	*δ*_H_ (*J* in Hz)
2	154.0	8.24, s	154.0	8.24, s	154.0	8.23, s	154.0	8.22, s	149.6	8.03, s	149.6	8.03, s	150.0	8.08, s
3	124.2		124.2		123.7		123.6		124.6		124.6		124.7	
4	181.7		181.7		182.0		182.0		173.6		173.6		173.6	
5	158.5		158.6		155.8		155.9		154.0		154.0		153.7	
6	110.2		110.2		109.5		109.4		100.3		100.2		105.1	
7	158.3		158.3		166.2		166.0		162.2		162.2		154.2	
8	99.2		99.3		100.5		100.5		103.5		103.4		100.7	
9	154.5		154.4		156.7		156.9		152.3		152.3		151.6	
10	106.2		106.2		106.9		106.9		107.9		108.0		108.1	
OH-5		13.20, s		13.20, s		13.20, s		13.21, s						
1′	123.2		123.2		123.1		123.1		122.9		122.9		122.7	
2′,6′	131.2	7.46, d (8.5)	131.2	7.46, d (8.5)	131.1	7.47, d (8.5)	131.1	7.47, d (8.5)	130.3	7.27, d (8.5)	130.3	7.27, d (8.5)	130.2	7.29, d (8.5)
3′,5′	115.9	6.91, d (8.5)	115.9	6.91, d (8.5)	115.9	6.90, d (8.5)	115.9	6.90, d (8.5)	114.6	6.77, d (8.5)	114.6	6.77, d (8.5)	114.6	6.78, d (8.5)
4′	158.4		158.3		158.4		158.3		156.8		156.8		156.9	
OH-4′														9.44, s
1′′	30.0	2.97, dd (6.5, 13.0) 2.86, dd (7.0, 13.0)	29.9	2.97, dd (6.5, 13.0) 2.86, dd (7.0, 13.0)	27.4	3.19, 2H, m	27.4	3.18, 2H, m	25.7	2.82, dd (5.5, 16.5) 2.42, dd (7.5, 16.5)	25.7	2.77, dd (5.5, 17.0) 2.47, dd (7.0, 17.0)	25.6	2.78, dd (5.5, 17.0) 2.42, dd (7.5, 17.0)
2′′	75.3	4.39, t (7.0Hz)	75.3	4.39, t (7.0Hz)	92.2	4.82, t (8.5)	92.3	4.84, dd (7.5, 9.5)	66.5	3.64, td (5.0, 7.5)	66.4	3.65, q (6.0)	66.5	3.65, td (5.5, 7.5)
3′′	149.2		149.2		71.6		71.5		77.9		77.8		78.0	
4′′	110.3	4.73, brs 4.64, brs	110.3	4.73, brs 4.63, brs	25.5	1.25, s	26.0	1.27, s	25.5	1.31, s	25.3	1.28, s	20.5	1.19, s
5′′	17.7	1.83, s	17.8	1.83, s	25.6	1.32, s	25.2	1.29, s	20.2	1.18, s	20.7	1.20, s	25.3	1.30, s
OH-2′′										5.18, d (5.0)		5.16, d (4.5)		5.17, d (5.0)
1′′′	26.0	3.07, dd (5.5, 16.5) 2.73, dd (7.5, 16.5)	26.0	3.07, dd (5.5, 16.5) 2.73, dd (7.5, 16.5)	30.4	2.95, 2H, m	30.4	2.98, 2H, m	26.8	3.20, 2H, d (8.5)	26.8	3.20, 2H, d (8.0)	114.5	6.68, d (10.0)
2′′′	68.6	3.89, dd (5.5, 7.0)	68.7	3.89, dd (5.0, 7.5)	75.2	4.38, t (6.5)	74.9	4.45, t (6.5)	90.9	4.76, t (8.5)	90.9	4.75, t (8.5)	127.4	5.73, d (10.0)
3′′′	79.8		79.9		148.9		148.9		70.1		70.0		77.7	
4′′′	21.3	1.35, s	20.9	1.35, s	110.6	4.69, s 4.79, s	110.5	4.69, s 4.79, s	24.8	1.18, s	24.7	1.16, s	27.7	1.43, s
5′′′	25.7	1.45, s	25.9	1.45, s	17.6	1.84, s	17.9	1.84, s	25.6	1.15, s	25.8	1.17, s	27.8	1.45, s

**Figure 2:**
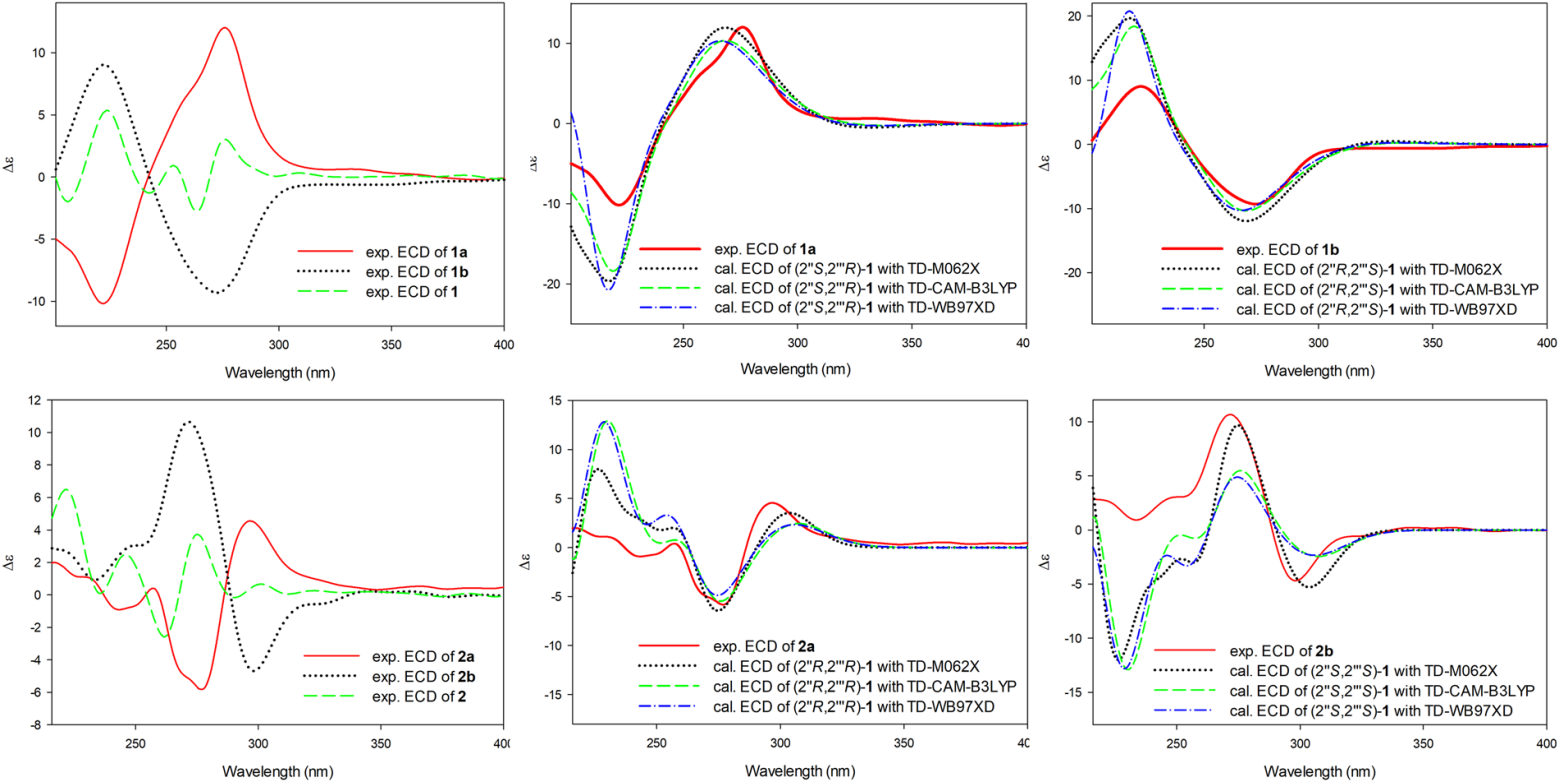
Experimental and calculated ECD spectra of **1** and **2** in acetonitrile.

Initially, due to the positive of its specific rotation {[α]^24^_D_ +4.3 (*c* 0.01, MeOH)} together with the detection of Cotton effects (CE) in the ECD spectrum (Fig. 2), **1** was supposed to be an optically pure compound. Therefore, a modified Mosher’s experiment was carried out to establish the absolute configurations at the C-2′′ and C-2′′′ positions^20^. Interestingly, when the (*R*) and (*S*)-MTPA esters of **1** were subjected to RP-C_18_ HPLC, two pairs of diastereomers including (*S*)-MTPA-**1a**/(*S*)-MTPA-**1b** and (*R*)-MTPA-**1a**/(*R*)-MTPA-**1b** were observed (Supplementary S.4), suggesting the racemic nature of 1. This suggestion was further confirmed by the detection of two peaks in the chiral HPLC analysis of 1. The enantiomeric separation of 1 by chiral HPLC let to the isolation of the enantiomers **1a** (*t*_R_ 11.14 min, [α]^22^_D_ +12.7) and **1b** (*t*_R_ 14.49 min, [α]^22^_D_ -28.7) (Supplementary S.23), which exhibited the mirror image-like ECD curves (Fig. 2).

The molecular formula of compound **2** was C_25_H_26_O_7_ (HRESIMS, *m/z* 439.1741 [M + H]^+^). The analyzing 1D and 2D NMR data of **2** indicated that **2** was a stereoisomer of **1**. Although CE curves were detected in the ECD spectrum of **2** (Fig. 2) along with a measurable optical rotation ([α]^24^_D_ +2.1), its racemic nature was demonstrated based on chiral HPLC analysis. Further enantiomer separation using chiral HPLC resulted in the isolation of enantiomers 2a (*t*_R_ 21.48 min, [α]^22^_D_ −26.2) and **2b** (*t*_R_ 23.52 min, [α]^22^_D_ +12.0) (Supplementary S.23).

In order to determine the absolute configurations of the enantiomers **1a** and **1b**, as well as **2a** and **2b**, quantum chemical ECD calculations were carried out and the results were compared with the experimental data. Four possible stereoisomers based on differences at the C-2′′ and C-2′′′ positions of the gross structure were built and separately subjected to a Merck molecular force field (MMFF) conformation search, followed by geometry optimization in density functional methods. The ECD data of the selected conformers were calculated using the time-dependent DFT (TDDFT) method.

As shown in Fig. 2, the calculated ECD spectra for the (2′′*S*,2′′′*R*) and (2′′*R*,2′′′*S*)-isomers were well matched with the experimental spectra of **1a** and **1b**, respectively, and the simulated spectra for the (2′′*R*,2′′′*R*) and (2′′*S*,2′′′*S*)-isomers were highly consistent with the experimental spectra of **2a** and **2b**, respectively. Besides, in order to further confirm the results, the additional ECD calculations were carried out using the CAM-B3LYP and WB97XD functionals, which yielded consistent ECD results (Fig. 2). On this basis, the absolute configurations of **1a**, **1b**, **2a**, and **2b** were assigned as depicted, which were named as (2′′*S*,2′′′*R*)-cudraisoflavone U, (2′′*R*,2′′′*S*)-cudraisoflavone U, (2′′*R*,2′′′*R*)-cudraisoflavone U, and (2′′*S*,2′′′*S*)-cudraisoflavone U, respectively.

The HRESIMS of compound **3** was indicated the molecular formula of C_25_H_26_O_7_ (*m/z* 439.1753 [M + H]^+^). Its 1D NMR spectra were similar to those of cudraisoflavone E^6^. In opposition to cudraisoflavone E, the HMBC correlations H-1′′′ [*δ*_H_ 2.95 (2H, m)]/C-7 (*δ*_C_ 166.2), C-8 (*δ*_C_ 100.5), and C-9 (*δ*_C_ 156.7) as well as the H-1′′ [*δ*_H_ 3.19 (2H, m)]/C-5 (*δ*_C_ 155.8), C-6 (*δ*_C_ 109.5) and C-7 revealed that the 2-hydroxyl-3-methylbut-3-enyl and 2-(1-hydroxy-1-methylethyl)dihydrofuran groups were located at the C-8 position, as well as the C-6 and C-7 positions, respectively. Thus, compound **3** was elucidated and named cudraisoflavone V.

**Figure 3:**
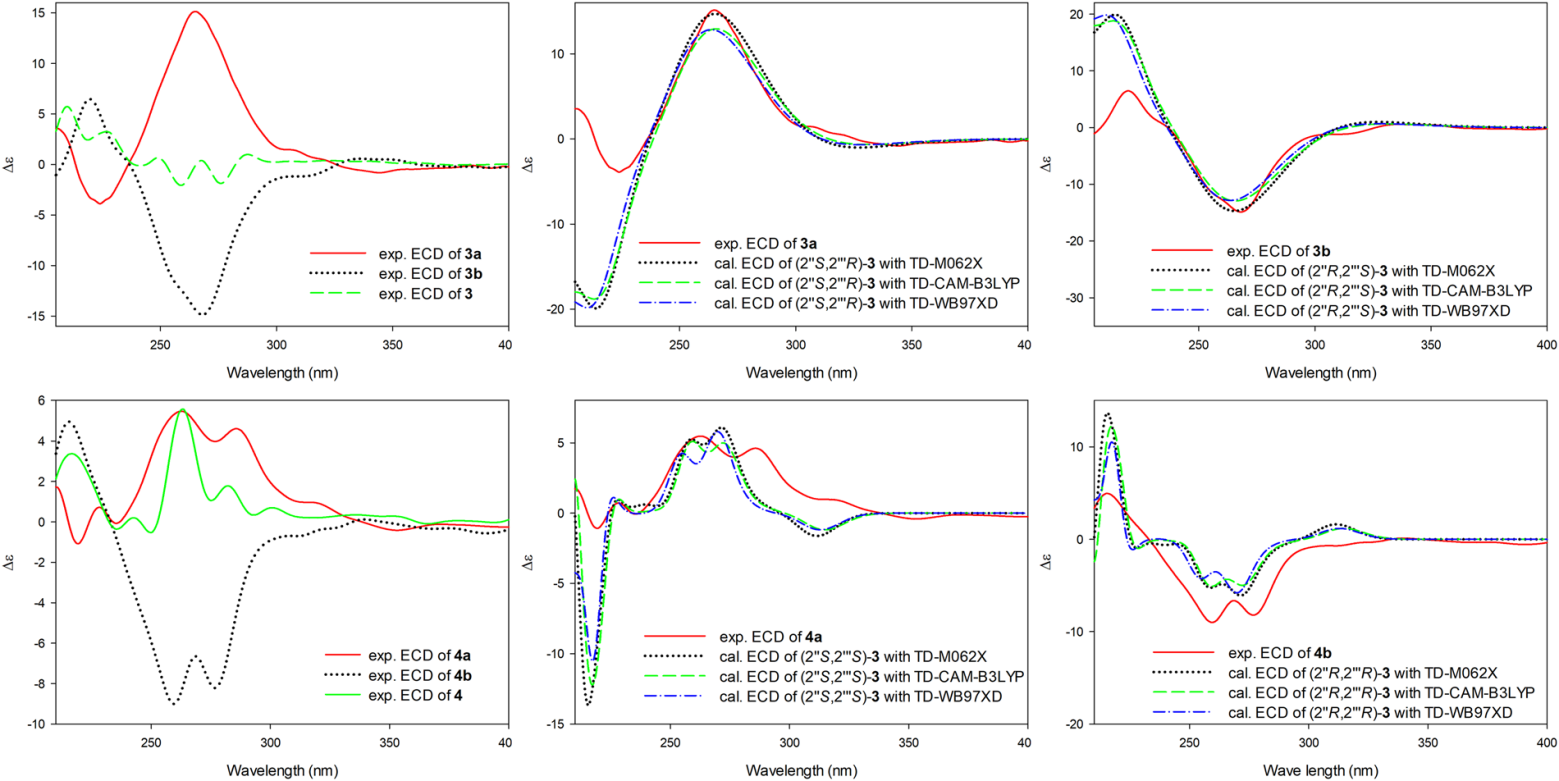
Experimental and calculated ECD spectra of **3** and **4** in acetonitrile.

In additionally, compound **3** was also established to be a racemic mixture due to the lack of CE curves, and further separated into **3a** (*t*_R_ 14.70 min) and **3b** (*t*_R_ 27.68 min) by chiral HPLC (Supplementary S.23). **3a** and **3b** displayed mirror image-like ECD curves (Fig. 3) and opposite specific rotations (**3a**: [α]^22^_D_ +16.2 and **3b**: [α]^22^_D_ −6.2).

The HRESIMS spectrum of compound **4** exhibited [M + H]^+^ signal at *m/z* 439.1754 (calcd. for C_25_H_27_O_7_, 439.1757), suggesting molecular formula of C_25_H_26_O_7_. The similarity of the NMR data (1D and 2D) of **4** and **3** demonstrated that **4** was a stereoisomer of **3**. Considering the racemic nature of **3**, **4** was also purified via HPLC using a chiral column to afford a pair of enantiomers 4a (*t*_R_ 15.13 min, [α]^22^_D_ +21.5) and **4b** (*t*_R_ 16.36 min, [α]^22^_D_ −22.5) (Supplementary S.23), which showed antipodal ECD curves (Fig. 3).

Quantum ECD calculations were also applied to measure the absolute configuration of **3a**, **3b**, **4a**, and **4b**. The measured spectra of **3a**, **3b**, **4a**, and **4b** fit well with the calculated ECD spectra for the (2′′*S*,2′′′*R*), (2′′*R*,2′′′*S*), (2′′*S*,2′′′*S*), and (2′′*R*,2′′′*R*)-isomers, respectively (Fig. 3), and the absolute configurations of **3a**, **3b**, **4a**, and **4b** were thus assigned as follows: (2′′*S*,2′′′*R*)-cudraisoflavone V, (2′′*R*,2′′′*S*)-cudraisoflavone V, (2′′*S*,2′′′*S*)-cudraisoflavone V, and (2′′*R*,2′′′*R*)-cudraisoflavone V, respectively.

The formula of compound **5** was established as C_25_H_26_O_7_ by the HRESIMS ion [M + H]^+^ at *m/z* 439.1740 (calcd. for C_25_H_27_O_7_, 439.1757). The 1D NMR spectra resembled those of cudraisoflavone I (Supplementary S.24)^6^. However, they differed in the presence of a 2-(1-hydroxy-1-methylethyl)dihydrofuran group [*δ*_H_ 3.20 (2 H, d, *J* = 8.5 Hz, H-1′′′), 4.76 (1 H, t, *J* = 8.5 Hz, H-2′′′), 1.18 (3 H, s, Me-4′′′), and 1.15 (3 H, s, Me-5′′′)] at the C-7 and C-8 positions instead of the furan group, confirmed by the HMBC cross-peaks H-1′′′/C-7 (*δ*_C_ 162.2), C-8 (*δ*_C_ 103.5) and C-9 (*δ*_C_ 152.3). Based on these, the structure of compound **5** was determined to be cudraisoflavone W.

**Figure 4:**
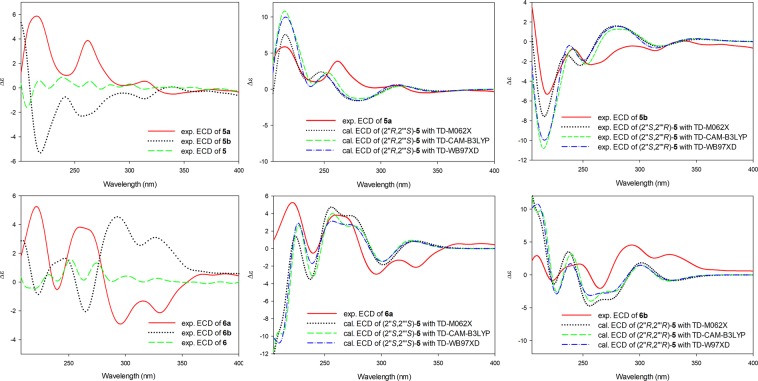
Experimental and calculated ECD spectra of **5** and **6** in acetonitrile.

The HRESIMS spectra of **6** resulted as the same molecular formula as that of **5**. It was a stereoisomer of **5**, as elucidated directly from the 1D and 2D NMR spectra. Additionally, no CE curves were detected in the ECD spectra of **5** and **6** (Fig. 4), indicating that these compounds were racemic mixtures, respectively. The racemic nature of **5** and **6** was also confirmed by chiral HPLC analysis. The further purification of **5** and **6** achieved of two pairs of enantiomers **5a** (*t*_R_ 8.06 min, [α]^22^_D_ +15.7) and **5b** (*t*_R_ 10.16 min, [α]^22^_D_ −11.2) as well as **6a** (*t*_R_ 12.95 min, [α]^22^_D_ +12.0) and **6b** (*t*_R_ 20.36 min, [α]^22^_D_ −10.7) (Supplementary S.23), respectively.

Similar to the case for **1–4**, the experimental ECD spectra of **5a**, **5b**, **6**a, and **6b** were highly consistent with the calculated ECD spectra of the (2′′*R*,2′′′S), (2′′*S*,2′′′*R*), (2′′*S*, 2′′′*S*), and (2′′*R*,2′′′*R*)-isomers, respectively (Fig. 4). Consequently, the absolute configurations of **5a**, **5b**, **6a**, and **6b** were determined as shown [(2′′*R*,2′′′*S*)-cudraisoflavone W, (2′′*S*,2′′′*R*)-cudraisoflavone W, (2′′*S*,2′′′*S*)-cudraisoflavone W, and (2′′*R*,2′′′*R*)-cudraisoflavone W, respectively].

The molecular formula of compound **7** was C_25_H_24_O_6_ (HRESIMS). The ^1^H and ^13^C NMR signals closely matched those of **6**. However, they differed in the replacement of a 2-(1-hydroxy-1-methylethyl)dihydrofuran group by a 2,2-dimethylpyran group [*δ*_H_ 6.68 (1H, d, *J* = 10.0 Hz, H-1′′′), 5.73 (1H, d, *J* = 10.0 Hz, H-2′′′), 1.43 (3H, s, Me-4′′′), and 1.45 (3H, s, Me-5′′′)] at the C-7 and C-8 positions, confirmed by the HMBC correlations H-1′′′/C-7 (*δ*_C_ 154.2), C-8 (*δ*_C_ 100.7), and C-9 (*δ*_C_ 151.6). Thus, compound **7** was determined to be cudraisoflavone X.

**Figure 5:**
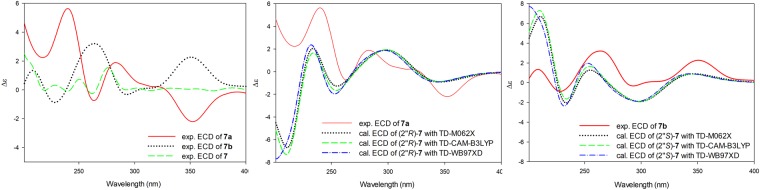
Experimental and calculated ECD spectra of **7** in acetonitrile.

Compound **7** was also found to be a racemic mixture due to the presence of two peaks in the chiral HPLC analysis. Further HPLC separation led to the isolation of two enantiomers **7a** (*t*_R_ 11.40 min, [α]^22^_D_ +18.0) and **7b** (*t*_R_ 18.58 min, [α]^22^_D_ −13.2) (Supplementary S.23). **7a** and **7b** were assigned as (2′′*R*)-cudraisoflavone X and (2′′*S*)-cudraisoflavone X, respectively, based on comparison of the experimental ECD spectral data with those of the (2′′*R*) and (2′′*S*)-isomers (Fig. 5).

The racemic compounds 1–7 were evaluated for neuroprotective activity against oxygen-glucose deprivation/reoxygenation (ODG/R)-induced neuronal cell death in SH-SY5Y cells. Of these, 1 exhibited a significant protective effect with an EC_50_ value of 5.5 µM (carnosine was used as a positive control, EC_50_ 13.4 µM) (Table 2)^21^. The rest of the compounds were inactive (EC_50_ > 20 µM). Accordingly, enantiomers **1a** and **1b** were further separately examined for their neuroprotective potential and both were found to attenuate ODG/R-induced neurotoxicity with EC_50_ values of 4.0 µM and 10.0 µM, respectively (Table 2).

**Table 2:** Neuroprotective and inhibitory of ROS generation activities of isolated compounds.

Compound	Protective effect against cell death (EC_50_, μM)	Inhibitory effect against ROS generation (IC_50_, μM)
**1**	5.5 ± 1.4^##^	6.9 ± 1.2^#^
**1a**	4.0 ± 1.0^###^	4.5 ± 2.5^##^
**1b**	10.0 ± 2.1	9.5 ± 3.2
**2**	>20	—^a^
**3**	>20	—^a^
**4**	>20	—^a^
**5**	>20	—^a^
**6**	>20	—^a^
**7**	>20	—^a^
Carnosine	13.4 ± 1.5	14.2 ± 2.3

EC_50_ and IC_50_ values were determined in a semi-logarithmic graph with 4 different concentrations. ^a^IC_50_ value not determined. (^#^p < 0.05, ^##^p < 0.01, and ^###^p < 0.001 versus carnosine, a control compound).

Moreover, although the causes of neurodegenerative diseases have not been clearly elucidated, many experimental evidences suggested that oxidative stress resulting in the generation of reactive oxygen species (ROS) plays a pivotal role in neurodegenerative diseases^16,17,22^. Furthermore, recent biological studies indicate that several isoflavones are beneficial for reducing oxidative stress in neurons and protecting against neurodegenerative diseases^22–25^. Consequently, the inhibitory effect of **1**, **1a**, and **1b** on the ODG/R-induced intracellular ROS generation in SH-5Y5Y cells was assessed. As shown in Table 2, **1**, **1a**, and **1b** inhibited ROS generation in ODG/R-induced SH-5Y5Y cells with IC_50_ values of 6.9 µM, 4.5 µM, and 9.5 µM, respectively.

Interestingly, **2** did not inhibit ODG/R-induced neuronal cell death although **2** has the same gross structure as that of **1**. On these grounds, it is suggested that the variety of stereochemistry has an apparent effect on the neuroprotective potential of these isoflavones. Recent study demonstrated that isoflavones from *M. tricuspidata* exerted neuroprotective activity via induction of Nox4-targeting miRNAs and inhibition of the MAPK signal cascade in *in vitro* and *in vivo* models of cerebral ischemia^26^.

Besides, recently studies indicated that ingested flavonoids are mostly metabolized in the small and large intestines, and liver, then enter the bloodstream and can reach the central nervous system (CNS) by transporting across the blood brain barrier (BBB)^27–29^. However, to date, the knowledge about their capacity of reaching the CNS remain insufficient and inconsistent. The degree to which flavonoids can enter the CNS is still a disagreement, in spite of several studies indicated their presence in brain tissue after oral administration^28,29^. Therefore, the knowledge regarding flavonoids transport across BBB and how this is regulated is crucial. Recent study reported that flavonoids might pass through the BBB by transmembrane diffusion, which is dependent on the degree of their lipophilicity^27,30,31^. Furthermore, the evaluations of transmembrane transport of different flavonoids such as genistein, (+)-catechin, hesperidin, and quercetin via blood-brain barrier cells models indicated that after treatment for 3 h, the obtained concentrations of these flavonoids were 3–10 µM, which was sufficient concentration to have beneficial effects^30,32–34^. In present study, isolated compounds from *M. tricuspidata* were genistein-based flavonoids, suggesting they may possess the ability to pass through BBB and reach the sufficient concentration.

Consequently, the isolated compounds from *M. tricuspidata* could be promising candidates for the treatment of cerebral ischemia and more investigations are needed to understand their cellular mechanisms of action in the brain for fully exploring their neuroprotective potential.

## Methods

### General experimental procedures

IR spectra were recorded on a Varian 640-IR spectrometer. Optical rotation was measured on a JASCO P-2000 polarimeter. UV spectra were recorded on an OPTIZEN POP spectrophotometer. ECD measurements were performed using a JASCO J-1100 spectrometer. 1D and 2D NMR spectra were measured on a Varian VNMRS 500 MHz system. HRESIMS data were obtained on a Waters Q-TOF micromass spectrometer. Column chromatography (CC) was carried out using Kieselgel 60 silica gel (40–60 μm, 70–230 mesh, Merck) and reverse-phase (RP) C_18_ silica gel (12 μm, YMC, Kyoto, Japan). The HPLC system consisted of a Varian Prostar 210 system, a YMC J′sphere ODS-H80 column (10 × 250 mm, 4 μm, YMC Co., Ltd., Kyoto, Japan), along with Chiralpak IA and IB columns (4.6 × 250 mm, 5 μm, Daicel, Osaka, Japan).

### Plant materials

The collection of fruits of *Maclura tricuspidata* and deposition of voucher specimen (KH1-5-090904) were carried out as previously described^7^.

### Extraction and Isolation

Fresh fruits of *M. tricuspidata* (10.7 kg) were extracted in 100% MeOH (3 × 10 L) at room temperature over the course of ten days. The extracts were concentrated under vacuum to afford a residue (TH1-1-1, 630.9 g), which was further extracted with *n*-hexane (48.43 g) and EtOAc (27.8 g).

The EtOAc fraction (TH1-2-2, 27.8 g) was fractionated by silica gel CC using CHCl_3_–MeOH (1:0 to 1:1) to give six fractions (TH1-4-1–TH1-4-6). Fraction TH1-4-3 (9.68 g) was further separated with a silica gel CC eluted with *n*-hexane–EtOAc (1:0 to 0:1) to generate seven subfractions (TH1-10-1–TH1-10-7). Fraction TH1-10-4 (4.7 g) was passed over silica gel CC using *n*-hexane–CHCl_3_–MeOH (1:0:0 to 0:1:1). Fraction TH1-74-12 (166.3 mg) was further separated into six subfractions (TH3-9-1–TH3-9-6) on RP-C_18_ silica gel CC using MeOH–H_2_O (1:1 to 10:0). Fraction TH3-9-1 (71.1 mg) was passed over silica gel CC using *n*-hexane–EtOAc (1:0 to 0:1), to obtain five fractions (TH3-19-1–TH3-19-5). The racemic mixtures **1** (5.1 mg), **2** (8.1 mg), **3** (4.1 mg), and **4** (6.3 mg) were obtained by preparative HPLC (MeOH–H_2_O, 60–81%, MeOH in H_2_O) of fraction TH3-19-3 (40.5 mg). Purification of mixtures **1** (Chiralpak IA; *n*-hexane–ethanol, 85:15), **2** (Chiralpak IB; *n*-hexane–ethanol, 90:10), **3** (Chiralpak IA; *n*-hexane–ethanol, 80:20), and **4** (Chiralpak IB; *n*-hexane–ethanol, 90:10) by chiral preparative HPLC afforded **1a** (1.3 mg, *t*_R_ 11.14 min), **1b** (1.4 mg, *t*_R_ 14.49 min), **2a** (1.6 mg, *t*_R_ 21.48 min), **2b** (1.9 mg, *t*_R_ 23.52 min), **3a** (1.1 mg, *t*_R_ 14.70 min), **3b** (1.4 mg, *t*_R_ 27.68 min), **4a** (1.5 mg, *t*_R_ 15.13 min), and **4b** (1.4 mg, *t*_R_ 16.36 min), respectively. Purification of fractions TH3-9-2 (24.4 mg) and TH3-9-3 (9.1 mg) via preparative HPLC (MeOH–H_2_O, 60–85%, MeOH in H_2_O) yielded the racemic mixture **7** (17.4 mg). The enantiomers **7a** (1.6 mg, *t*_R_ 11.40 min) and **7b** (1.6 mg, *t*_R_ 18.58 min) were obtained by chiral HPLC (Chiralpak IA; *n*-hexane–ethanol, 85:15). Fraction TH1-74-14 (240.4 mg) was separated into four subfractions TH3-3-1–TH3-3-4 with a RP-C_18_ silica gel CC using MeOH–H_2_O (1:1 to 8:2). Fraction TH3-3-2 (96.2 mg) was separated into the racemic mixtures 5 (14.7 mg) and 6 (7.4 mg) with preparative HPLC (MeOH–H_2_O, 55–75%). Further purification of mixtures **5** (Chiralpak IA; *n*-hexane–ethanol, 80:20) and **6** (Chiralpak IA; *n*-hexane–ethanol, 85:15) by chiral preparative HPLC afforded **5a** (1.6 mg, *t*_R_ 8.06 min), **5b** (1.6 mg, *t*_R_ 10.16 min), **6a** (1.8 mg, *t*_R_ 12.95 min), and **6b** (1.1 mg, *t*_R_ 20.36 min), respectively.

*Cudraisoflavone U* (***1***): Yellow oil; [α]^24^_D_ +4.3 (*c* 0.01, MeOH); UV (MeOH) λ_max_ nm (log ɛ): 213 (4.22), 271 (4.31); IR (ATR) ν_max_ cm^−1^: 3324 (>OH), 1649 (>C=O); ^1^H and ^13^C NMR data see Table 1; HRESIMS *m/z* 439.1742 [M + H]^+^ (calcd. for C_25_H_25_O_7_, 439.1757).

**1a**: [α]^22^_D_ +12.7 (*c* 0.04, MeOH); CD (*c* 0.6 mM, ACN) Δε −10.18 (222), +12.02 (276).

**1b**: [α]^22^_D_ −28.7 (*c* 0.04, MeOH); CD (*c* 0.6 mM, ACN) Δε +9.06 (221), −9.34 (272).

*Epi*-*cudraisoflavone U* (**2**): Yellow oil; [α]^24^_D_ +2.1 (*c* 0.01, MeOH); UV (MeOH) λ_max_ nm (log ɛ): 214 (4.33), 271 (4.41); IR (ATR) ν_max_ cm^−1^: 3324 (>OH), 1648 (>C=O); ^1^H and ^13^C NMR data see Table 1; HRESIMS *m/z* 439.1741 [M + H]^+^ (calcd.. for C_25_H_25_O_7_, 439.1757).

**2a**: [α]^22^_D_ −26.2 (*c* 0.04, MeOH); CD (*c* 0.6 mM, ACN) Δε −0.82 (244), +0.68 (257), −5.97 (275), +4.75 (297).

**2b**: [α]^22^_D_ +12.0 (*c* 0.04, MeOH); CD (*c* 0.6 mM, ACN) Δε + 0.89 (233), +10.64 (272), −4.58 (298).

*Cudraisoflavone V* (**3**): Yellow oil; [α]^22^_D_ −2.8 (*c* 0.01, MeOH); UV (MeOH) λ_max_ nm (log ɛ): 216 (4.37), 270 (4.48); IR (ATR) ν_max_ cm^−1^: 3286 (>OH), 1660 (>C=O); ^1^H and ^13^C NMR data see Table 1; HRESIMS *m/z* 439.1753 [M + H]^+^ (calcd. for C_25_H_27_O_7_, 439.1757).

**3a**: [α]^22^_D_ +16.2 (*c* 0.04, MeOH); CD (*c* 0.6 mM, ACN) Δε −4.01 (224), +15.28 (264), −1.01 (341).

**3b**: [α]^22^_D_ −6.2 (*c* 0.04, MeOH); CD (*c* 0.6 mM, ACN) Δε + 6,38 (220), −14.90 (268), +0.62 (338).

*Epi-cudraisoflavone V* (***4***): Yellow oil; [α]^22^_D_ −5.2 (*c* 0.01, MeOH); UV (MeOH) λ_max_ nm (log ɛ): 216 (4.35), 270 (4.48); IR (ATR) ν_max_ cm^−1^: 3327 (>OH), 1660 (>C=O); ^1^H and ^13^C NMR data see Table 1; HRESIMS *m/z* 439.1754 [M + H]^+^ (calcd. for C_25_H_27_O_7_, 439.1757).

**4a**: [α]^22^_D_ +21.5 (*c* 0.04, MeOH); CD (*c* 0.6 mM, ACN) Δε −1.13 (219), +0.70 (228), −0.10 (236), +5.47 (262), +4.01 (276), +4.54 (286), −0.53 (352).

**4b**: [α]^22^_D_ −22.5 (*c* 0.04, MeOH); CD (*c* 0.6 mM, ACN) Δε + 4.90 (215), −9.09 (260), −6.79 (268), −8.27 (277), +0.11 (399).

*Cudraisoflavone W* (***5***): Yellow oil; [α]^24^_D_ +3.1 (*c* 0.01, MeOH); UV (MeOH) λ_max_ nm (log ɛ): 213 (4.30), 263 (4.41); IR (ATR) ν_max_ cm^−1^: 3281 (>OH), 1639 (>C=O); ^1^H and ^13^C NMR data see Table 1; HRESIMS *m/z* 439.1740 [M + H]^+^ (calcd. for C_25_H_27_O_7_, 439.1757).

**5a**: [α]^22^_D_ +15.7 (*c* 0.04, MeOH); CD (*c* 0.6 mM, ACN) Δε + 5.81 (216), +1.02 (243), +3.80 (262), +0.14 (296), +0.43 (315), −0.58 (339).

**5b**: [α]^22^_D_ −11.2 (*c* 0.04, MeOH); CD (*c* 0.6 mM, ACN) Δε −5.30 (218), −6.71 (241), −2.36 (255), −0.38 (297), −0.91 (314), +0.19 (337).

*Epi-cudraisoflavone W* (**6**): Yellow oil; [α]^24^_D_ −3.2 (*c* 0.01, MeOH); UV (MeOH) λ_max_ nm (log ɛ): 214 (4.30), 263 (4.40); IR (ATR) ν_max_ cm^−1^: 3365 (>OH), 1640 (>C=O); ^1^H and ^13^C NMR data see Table 1; HRESIMS *m/z* 439.1744 [M + H]^+^ (calcd. for C_25_H_27_O_7_, 439.1757).

**6a**: [α]^22^_D_ +12.0 (*c* 0.04, MeOH); CD (*c* 0.6 mM, ACN) Δε + 5.23 (221), −0.60 (240), +3.91 (260), −2.95 (296), −1.29 (315), −2.26 (332), +0.48 (368).

**6b**: [α]^22^_D_ −10.7 (*c* 0.04, MeOH); CD (*c* 0.6 mM, ACN) Δε −0.91 (223), +1.66 (247), −2.05 (265), +4.53 (292), +2.52 (313), +2.97 (328).

*Cudraisoflavone X* (**7**): Yellow oil; [α]^24^_D_ −4.9 (*c* 0.01, MeOH); UV (MeOH) λ_max_ nm (log ɛ): 210 (4.33), 268 (4.64), 344 (3.63); IR (ATR) ν_max_ cm^−1^: 3318 (>OH), 1630 (>C=O); ^1^H and ^13^C NMR data see Table 1; HRESIMS *m/z* 419.1476 [M − H]^−^ (calcd. for C_25_H_23_O_6_, 419.1495).

**7a**: [α]^22^_D_ +18.0 (*c* 0.04, MeOH); CD (*c* 0.6 mM, ACN) Δε + 2.14 (218), +5.59 (240), −0.70 (263), +1.83 (283), −2.24 (352).

**7b**: [α]^22^_D_ −13.2 (*c* 0.04, MeOH); CD (*c* 0.6 mM, ACN) Δε + 1.33 (209), −0.91 (229), +3.17 (264), −0.28 (294), +2.24 (350).

### Computational details

The ECD calculations were performed as previously described with some modifications^7^. The DFT/B3LYP/cc-pTVZ level was employed for optimizing and calculating the relative energies of the initial low-energy conformers. Calculation of the ECD spectra were carried out at the TDDFT/M062X/def2TZVP level. Additional ECD calculations were performed using the CAM-B3LYP and WB97XD functionals in order to further confirm the calculated results.

### Measurement of cell viability and intracellular ROS and statistical analysis

The protective effects against ODG/R-induced cell death and intracellular ROS generation in SH-SY5Y cells of test compounds and statistical analysis were carried out as previously described^35^. All experimental data are expressed as the mean value ± standard deviation from three replicates for each experiment. Statistical significance between multiple groups was determined by one-way ANOVA (PRISM Graph Pad, San Diego, CA, USA). When the ANOVA showed a significant difference, Bonferroni’s multiple comparison *post hoc* test was conducted. P values less than 0.05 were regarded as statistically significant.

## Electronic supplementary material

Supplementary data for: Enantiomeric Isoflavones with neuroprotective activities from the Fruits of Maclura tricuspidata
