# Supplementary material for: Enantiomeric Isoflavones with neuroprotective activities from the Fruits of Maclura tricuspidata
Source: Sci Rep. 2019 Feb 11;9:1757. doi: 10.1038/s41598-018-36095-8 (PMC6370789; doi:10.1038/s41598-018-36095-8)
Supplement: Supplementary file 1 — Supplementary data for: Enantiomeric Isoflavones with neuroprotective activities from the Fruits of Maclura tricuspidata [file 41598_2018_36095_MOESM1_ESM.docx]

**Supplementary data for:**

Enantiomeric Isoflavones with neuroprotective activities from the Fruits of *Maclura tricuspidata*

*Nguyen Tuan Hiep,^a,b^ Jaeyoung Kwon,^c^ Sungeun Hong,^d^* *Nahyun Kim,^e^ Yuanqiang Guo,^f^ Bang Yeon Hwang,^g^ Woongchon Mar,^d,^* Dongho Lee^a,^**

*^a^*Department of Biosystems and Biotechnology, College of Life Science and Biotechnology, Korea University, Seoul 02841, Republic of Korea

*^b^*Department of Extraction Technology, Vietnam National Institute of Medicinal Materials, 3B Quang Trung, Hoan Kiem, Hanoi, Vietnam

*^c^*Natural Constituents Research Center, Korea Institute of Science and Technology (KIST), Gangneung 25451, Republic of Korea

*^d^*Natural Products Research Institute, College of Pharmacy, Seoul National University, Seoul 151-742, Republic of Korea

*^e^*Forest Medicinal Resources Research Center, National Institute of Forest Science, Yeongju 36040, Republic of Korea

*^f^*State Key Laboratory of Medicinal Chemical Biology, College of Pharmacy, and Tianjin Key Laboratory of Molecular Drug Research, Nankai University, Tianjin 300350, People’s Republic of China

*^g^*College of Pharmacy, Chungbuk National University, Cheongju 361-763, Republic of Korea

Corresponding authors:

*Tel.: +82-2-3290-3017; Fax: +82-2-953-0737; E-mail: dongholee@korea.ac.kr (D. Lee)

*Tel.: +82-2-880-2473; Fax: +82-2-888-9122; E-mail: mars@snu.ac.kr (W. Mar)

**Table of contents**

S.1. HRESIMS spectrum of **1** 3

S.2. ^1^H NMR spectrum of **1** (Acetone-*d*_6_, 500 MHz) 4

S.3. ^13^C NMR spectrum of **1** (Acetone-*d*_6_, 500 MHz) 5

S.4. HPLC chromatogram of (*R*)- and (*S*)-MTPA esters of **1** 6

S.5. HRESIMS spectrum of **2** 7

S.6. ^1^H NMR spectrum of **2** (Acetone-*d*_6_, 500 MHz) 8

S.7. ^13^C NMR spectrum of **2** (Acetone-*d*_6_, 500 MHz) 9

S.8. HRESIMS spectrum of **3** 10

S.9. ^1^H NMR spectrum of **3** (Acetone-*d*_6_, 500 MHz) 11

S.10. ^13^C NMR spectrum of **3** (Acetone-*d*_6_, 500 MHz) 12

S.11. HRESIMS spectrum of **4** 13

S.12. ^1^H NMR spectrum of **4** (Acetone-*d*_6_, 500 MHz) 14

S.13. ^13^C NMR spectrum of **4** (Acetone-*d*_6_, 500 MHz) 15

S.14. HRESIMS spectrum of **5** 16

S.15. ^1^H NMR spectrum of **5** (DMSO-*d*_6_, 500 MHz) 17

S.16. ^13^C NMR spectrum of **5** (DMSO-*d*_6_, 500 MHz) 18

S.17. HRESIMS spectrum of **6** 19

S.18. ^1^H NMR spectrum of **6** (DMSO-*d*_6_, 500 MHz) 20

S.19. ^13^C NMR spectrum of **6** (DMSO-*d*_6_, 500 MHz) 21

S.20. HRESIMS spectrum of **7** 22

S.21. ^1^H NMR spectrum of **7** (DMSO-*d*_6_, 500 MHz) 23

S.22. ^13^C NMR spectrum of **7** (DMSO-*d*_6_, 500 MHz) 24

S.23. Chiral HPLC separation profiles of **1a**/**1b**−**7a**/**7b** 25

S.24. Structures of Cudraisoflavone D and Cudraisoflavone I. 26

## HRESIMS spectrum of **1**


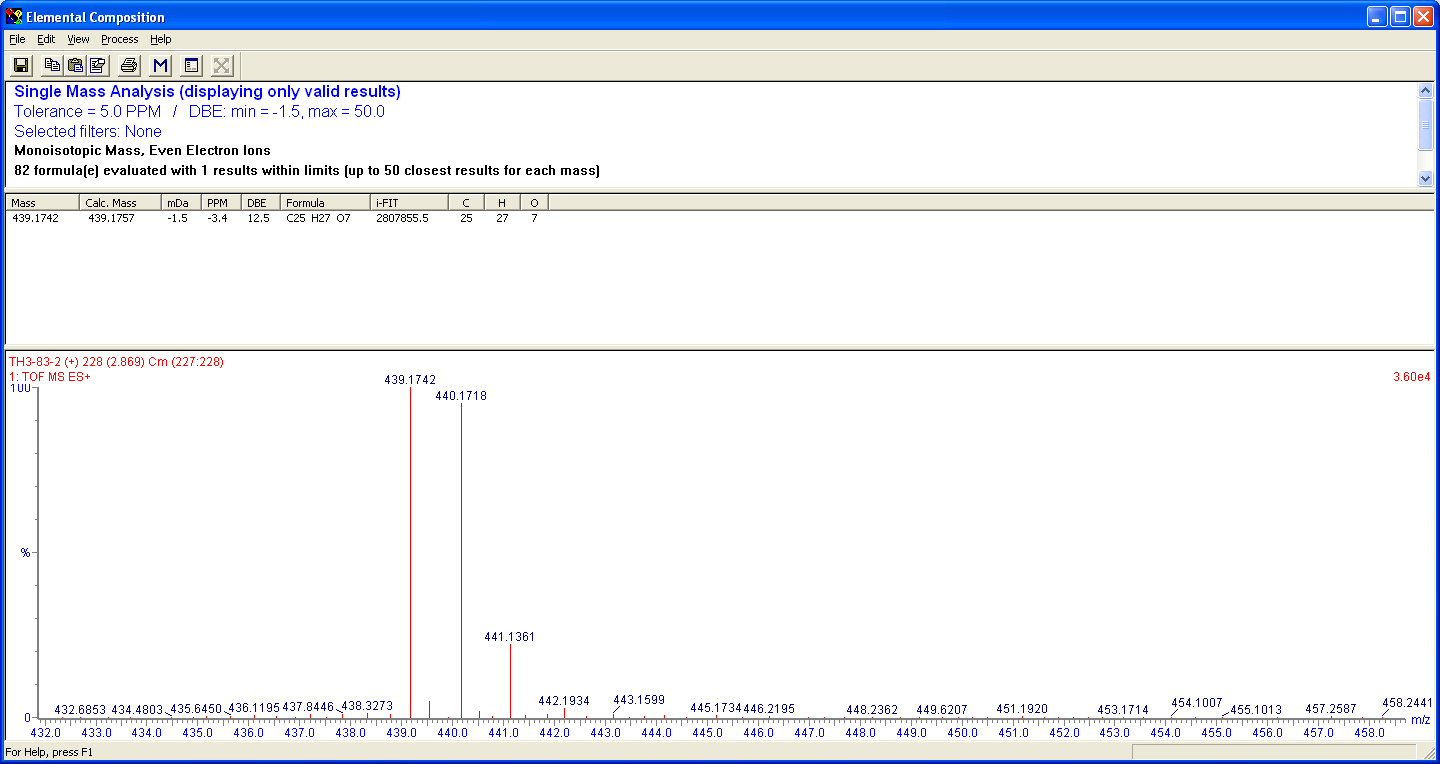


## ^1^H NMR spectrum of **1** (Acetone-*d*_6_, 500 MHz)

## ^13^C NMR spectrum of **1** (Acetone-*d*_6_, 500 MHz)

## HPLC chromatogram of (*R*)- and (*S*)-MTPA esters of **1**


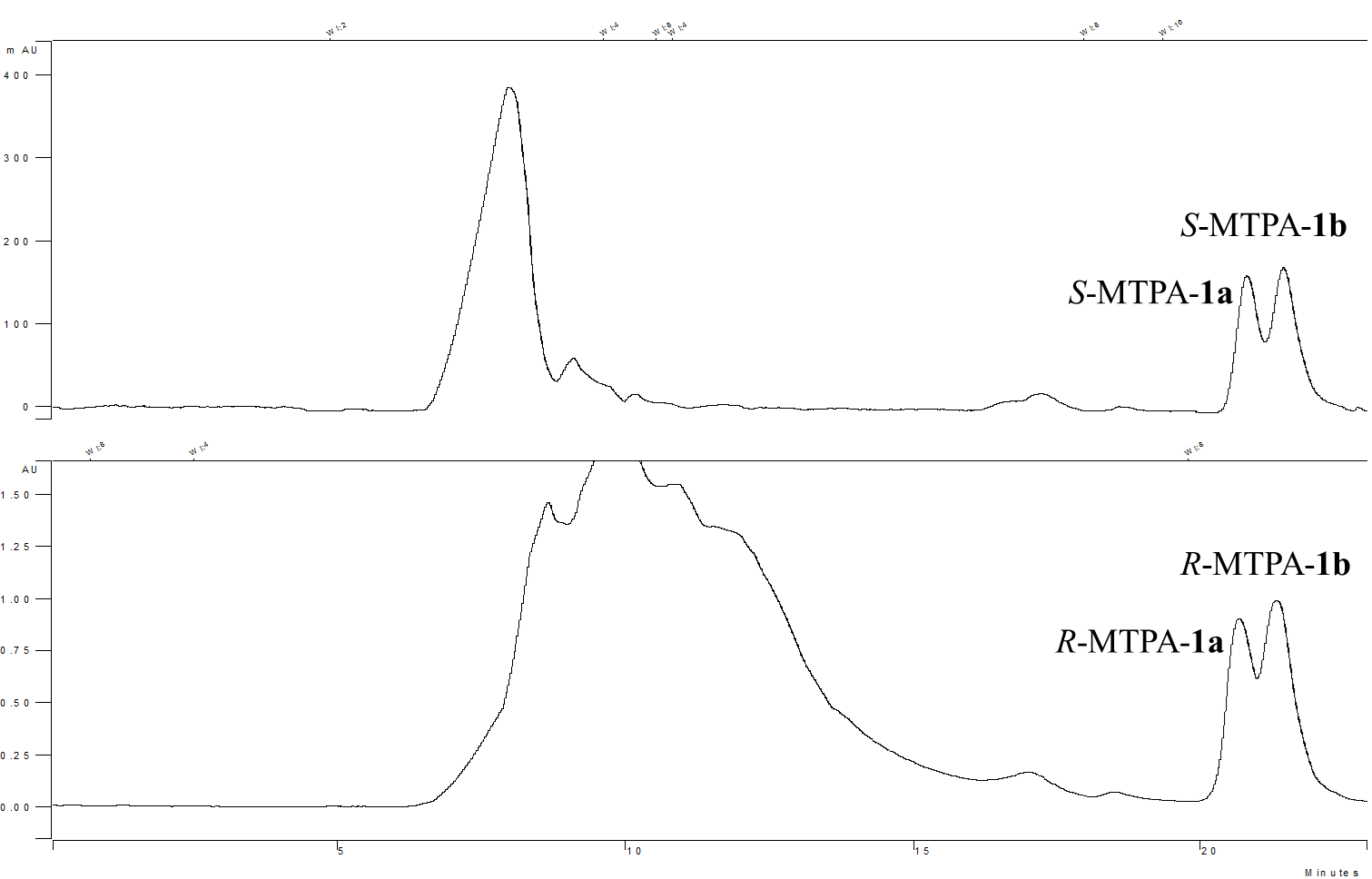


## HRESIMS spectrum of **2**


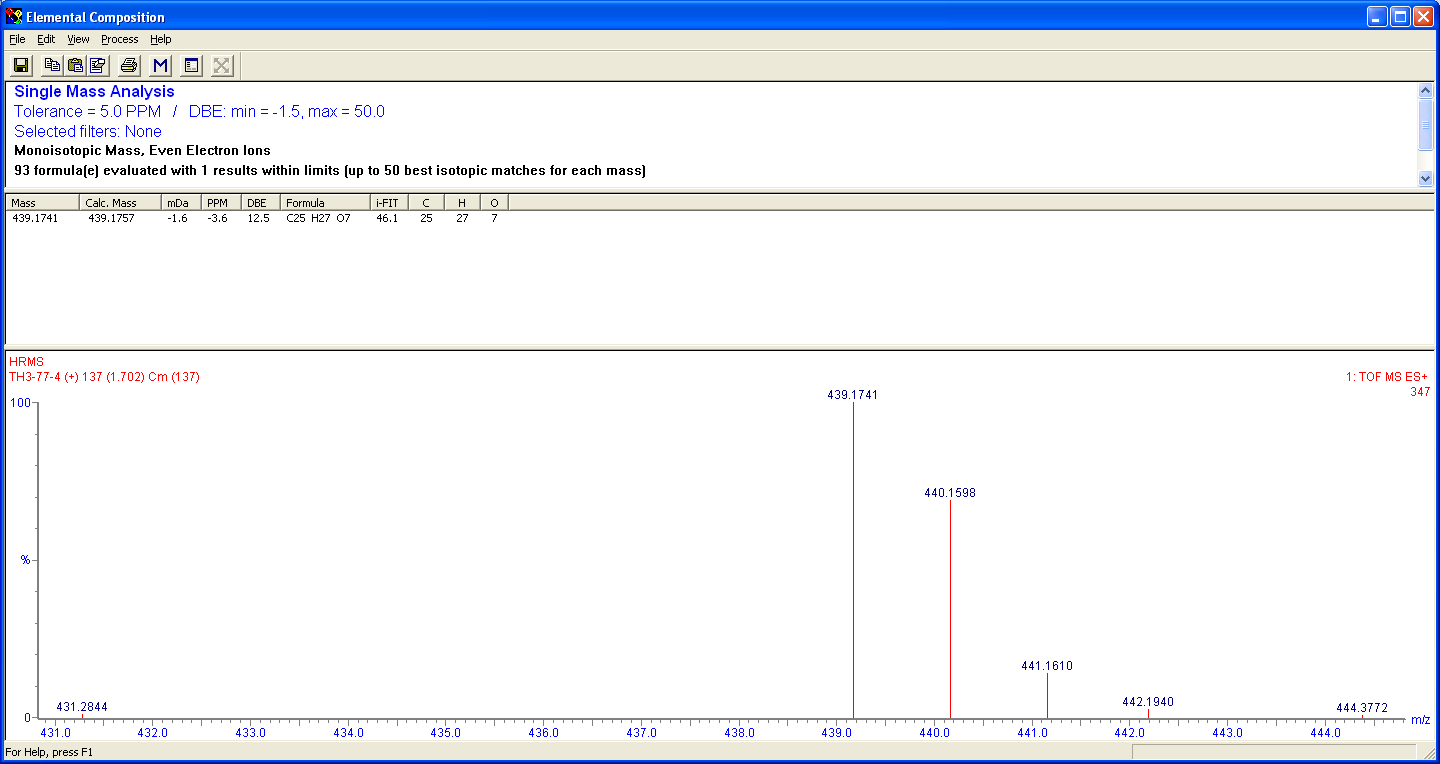


## ^1^H NMR spectrum of **2** (Acetone-*d*_6_, 500 MHz)

## ^13^C NMR spectrum of **2** (Acetone-*d*_6_, 500 MHz)

## HRESIMS spectrum of **3**


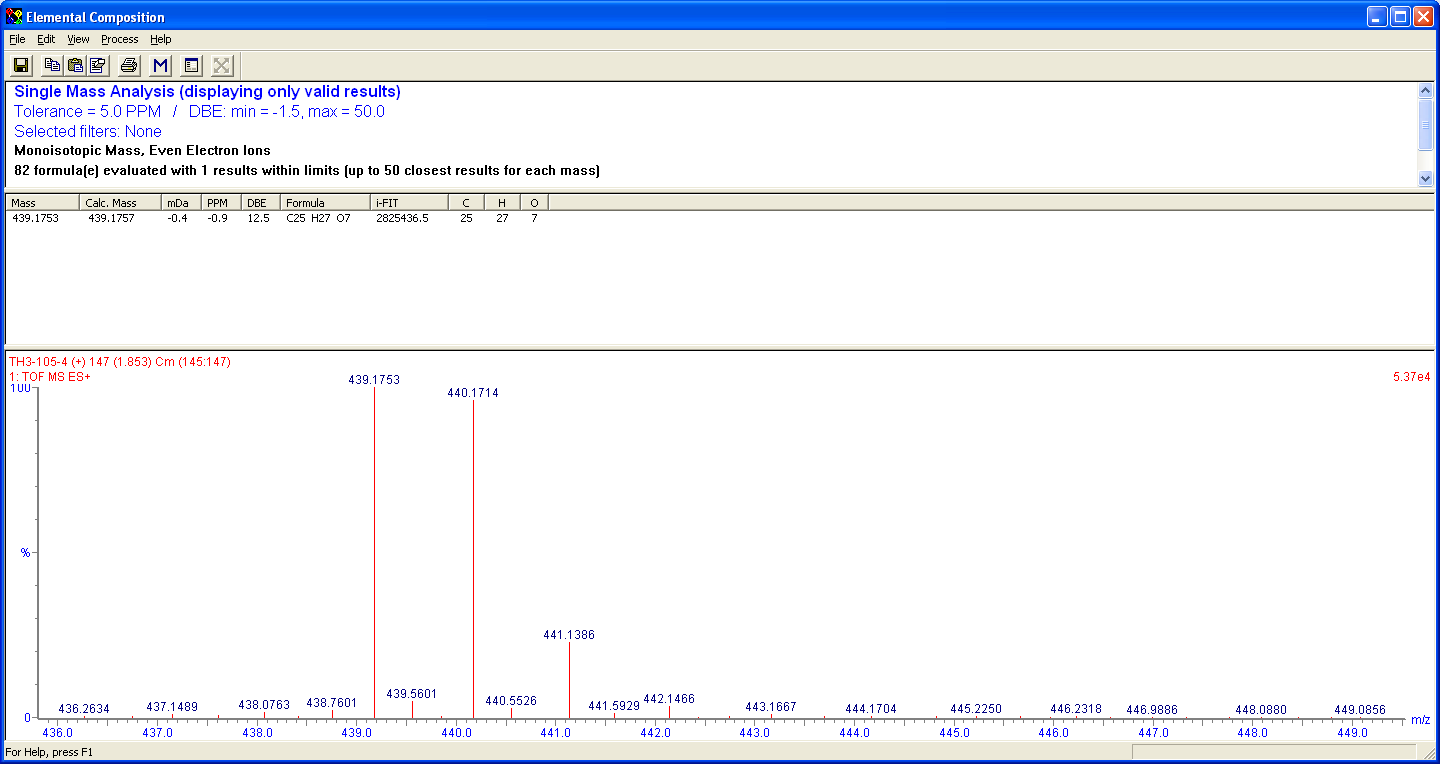


## ^1^H NMR spectrum of **3** (Acetone-*d*_6_, 500 MHz)

## ^13^C NMR spectrum of **3** (Acetone-*d*_6_, 500 MHz)

## HRESIMS spectrum of **4**


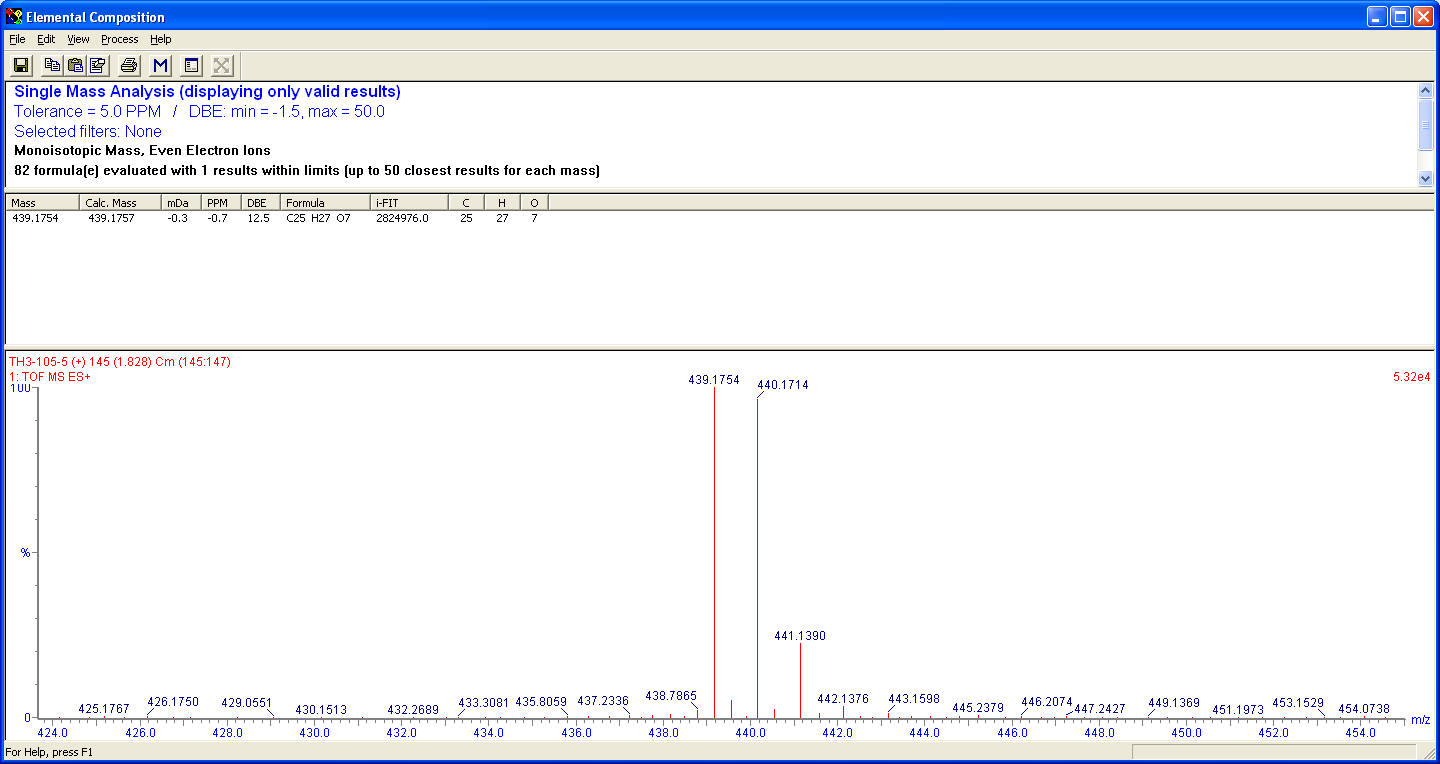


## ^1^H NMR spectrum of **4** (Acetone-*d*_6_, 500 MHz)

## ^13^C NMR spectrum of **4** (Acetone-*d*_6_, 500 MHz)

## HRESIMS spectrum of **5**


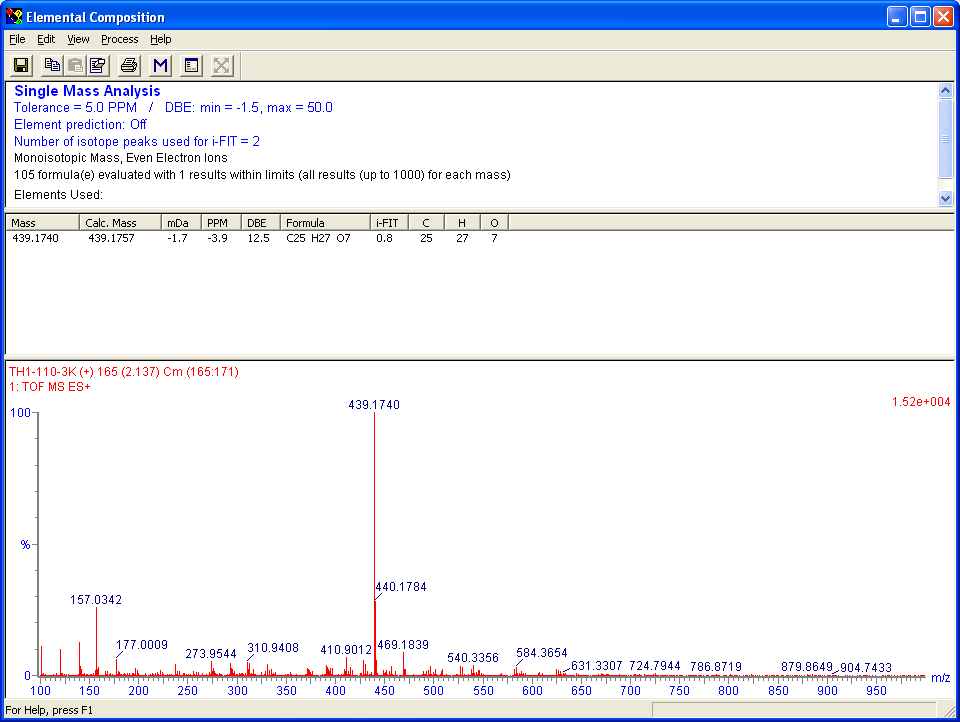


## ^1^H NMR spectrum of **5** (DMSO-*d*_6_, 500 MHz)

## ^13^C NMR spectrum of **5** (DMSO-*d*_6_, 500 MHz)

## HRESIMS spectrum of **6**


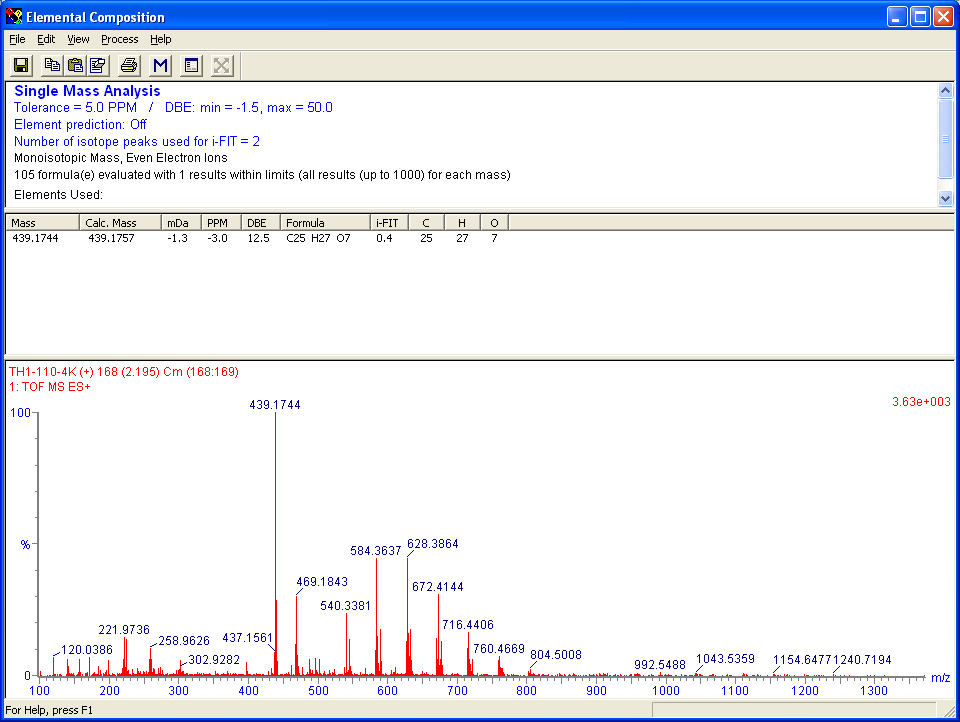


## ^1^H NMR spectrum of **6** (DMSO-*d*_6_, 500 MHz)

## ^13^C NMR spectrum of **6** (DMSO-*d*_6_, 500 MHz)

## HRESIMS spectrum of **7**


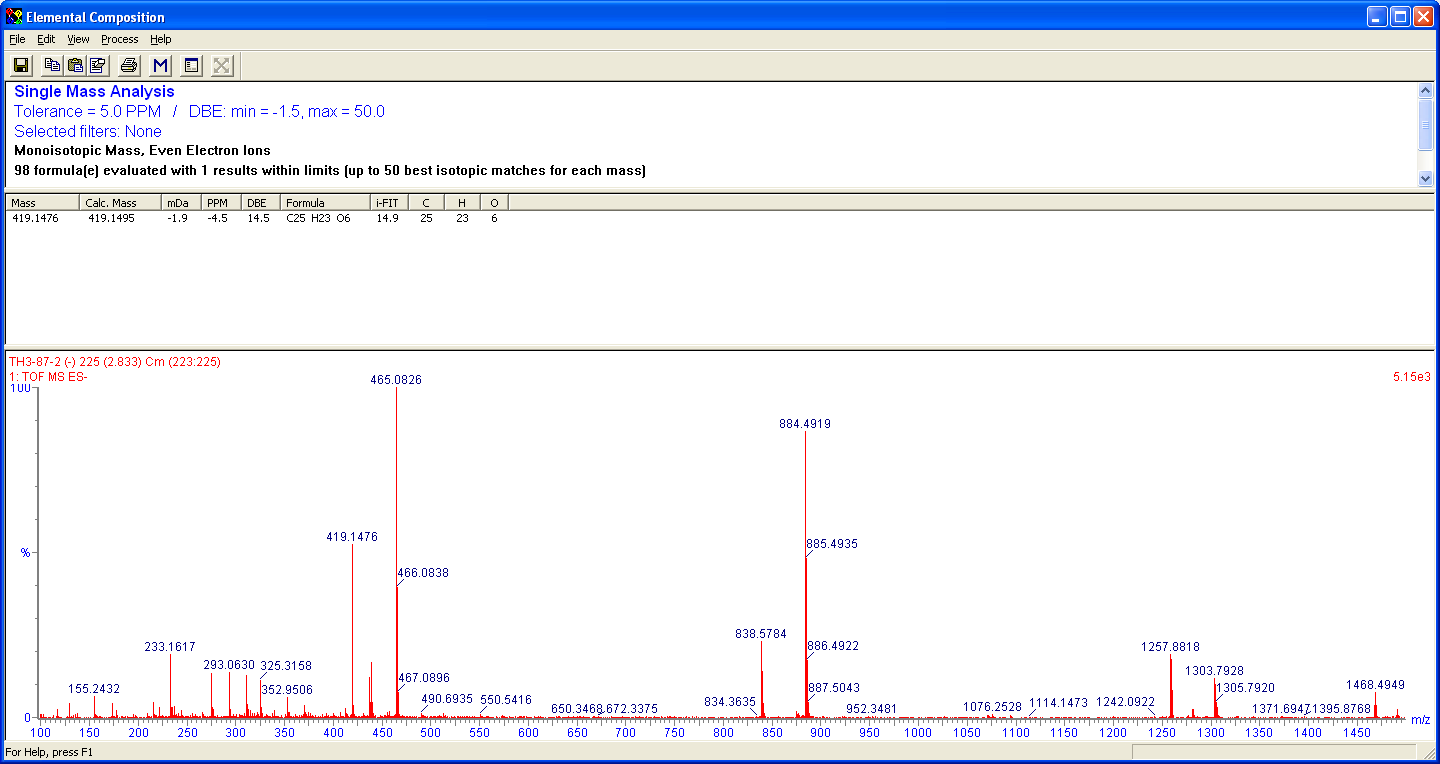


## ^1^H NMR spectrum of **7** (DMSO-*d*_6_, 500 MHz)

## ^13^C NMR spectrum of **7** (DMSO-*d*_6_, 500 MHz)

## S.23. Chiral HPLC separation profiles of **1a**/**1b**−**7a**/**7b**


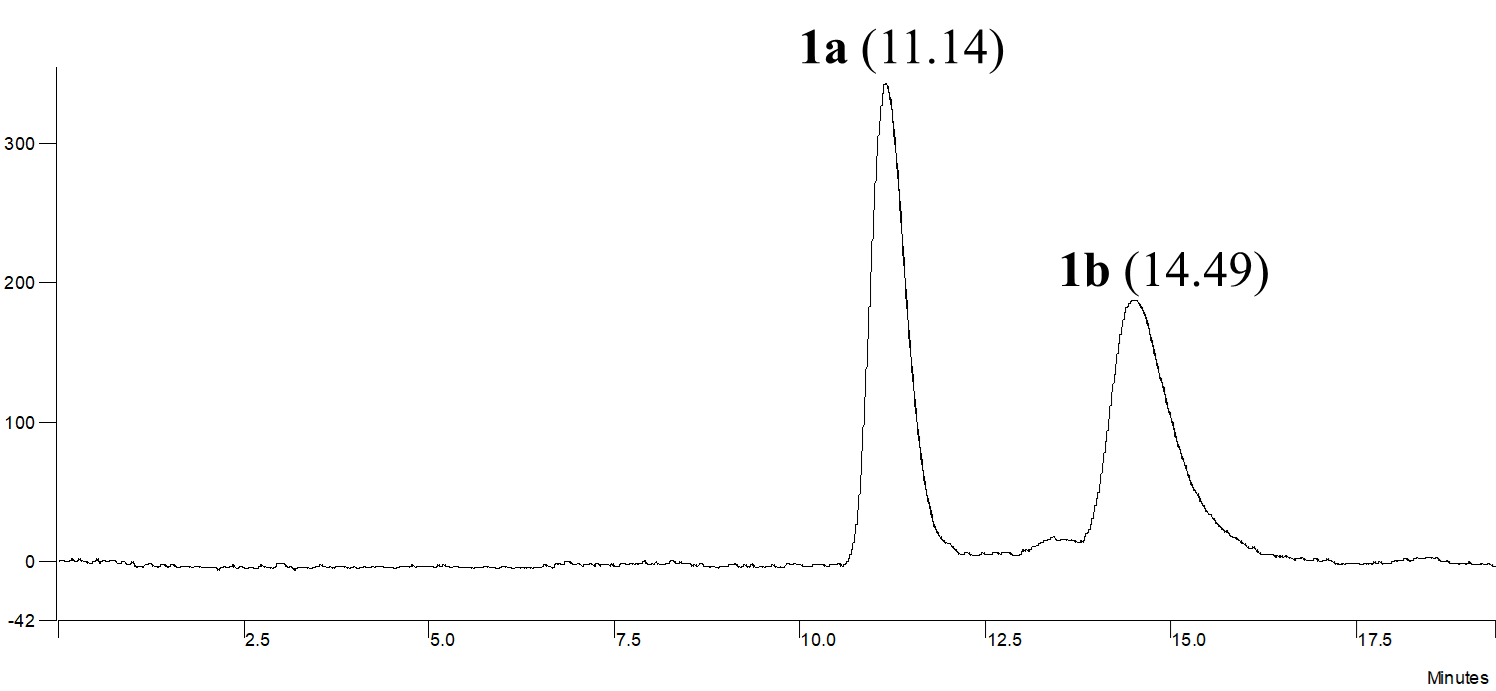

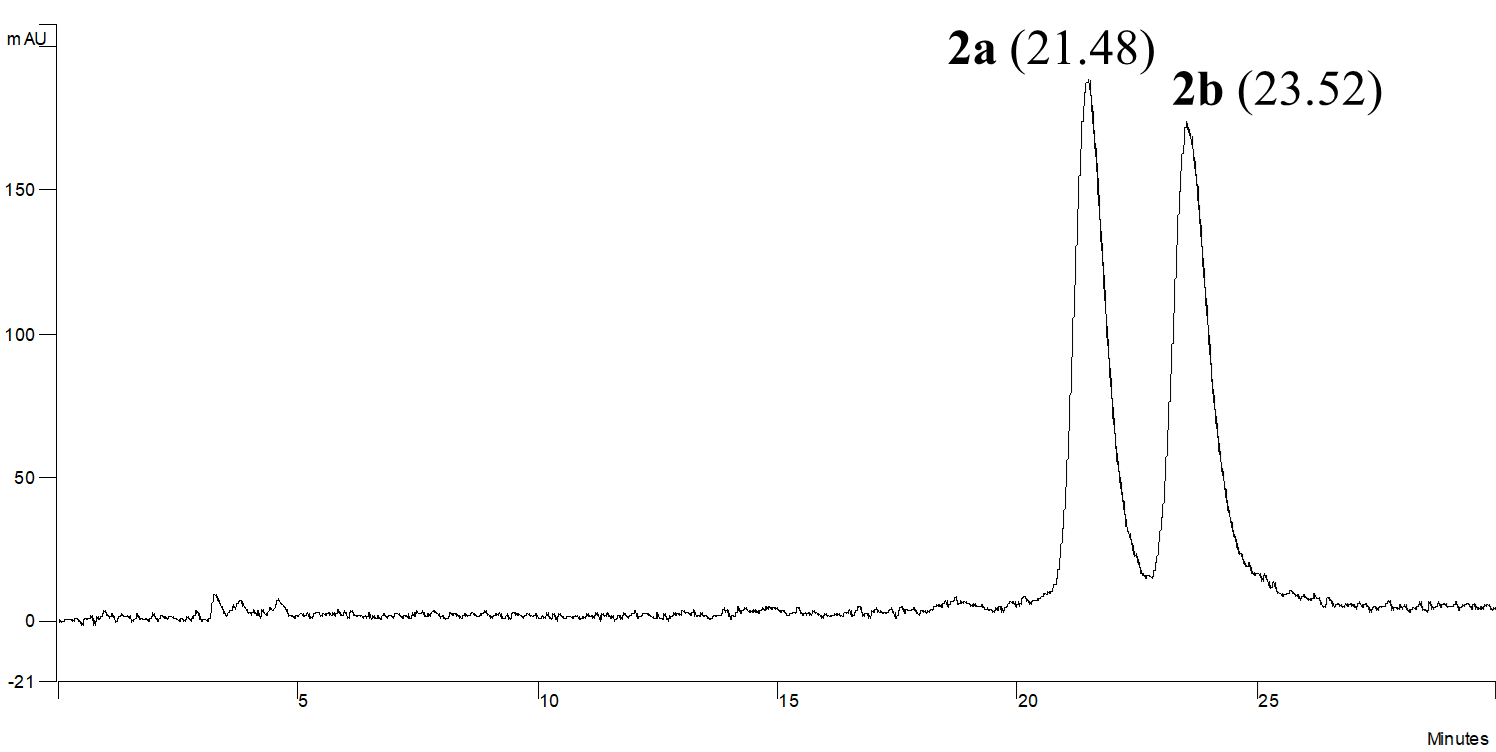

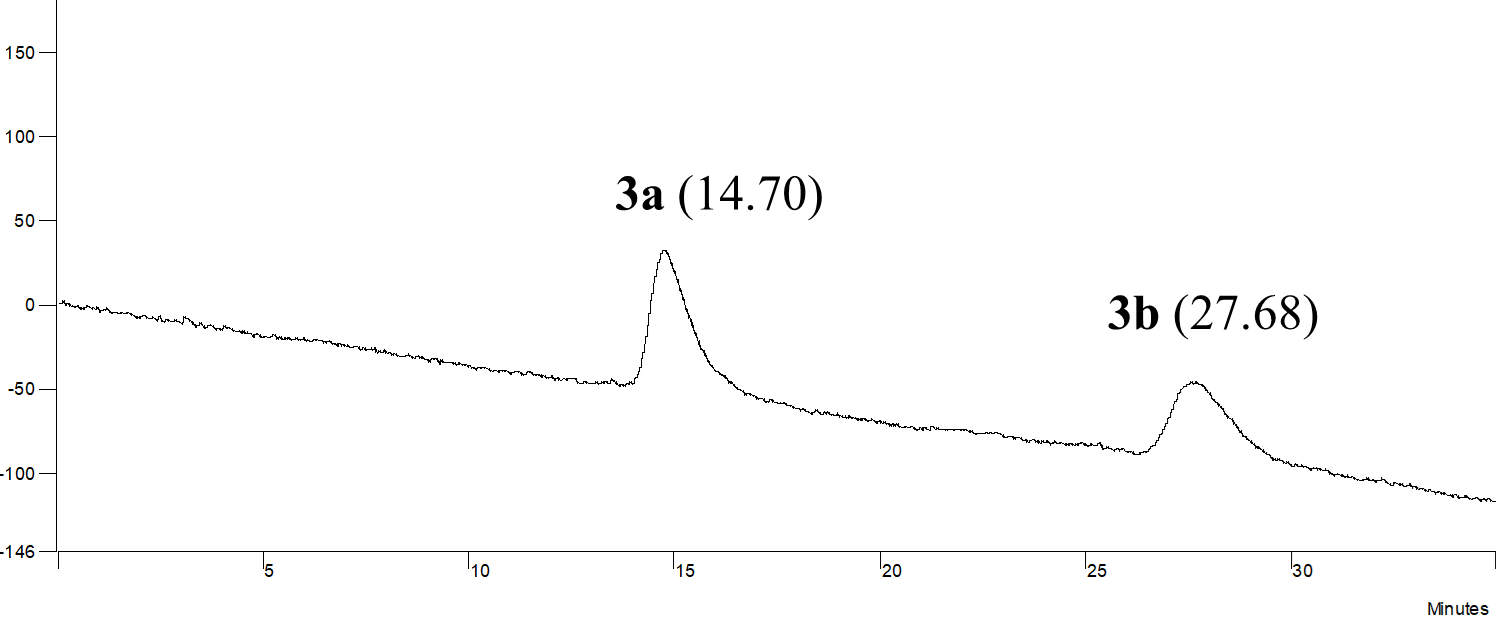

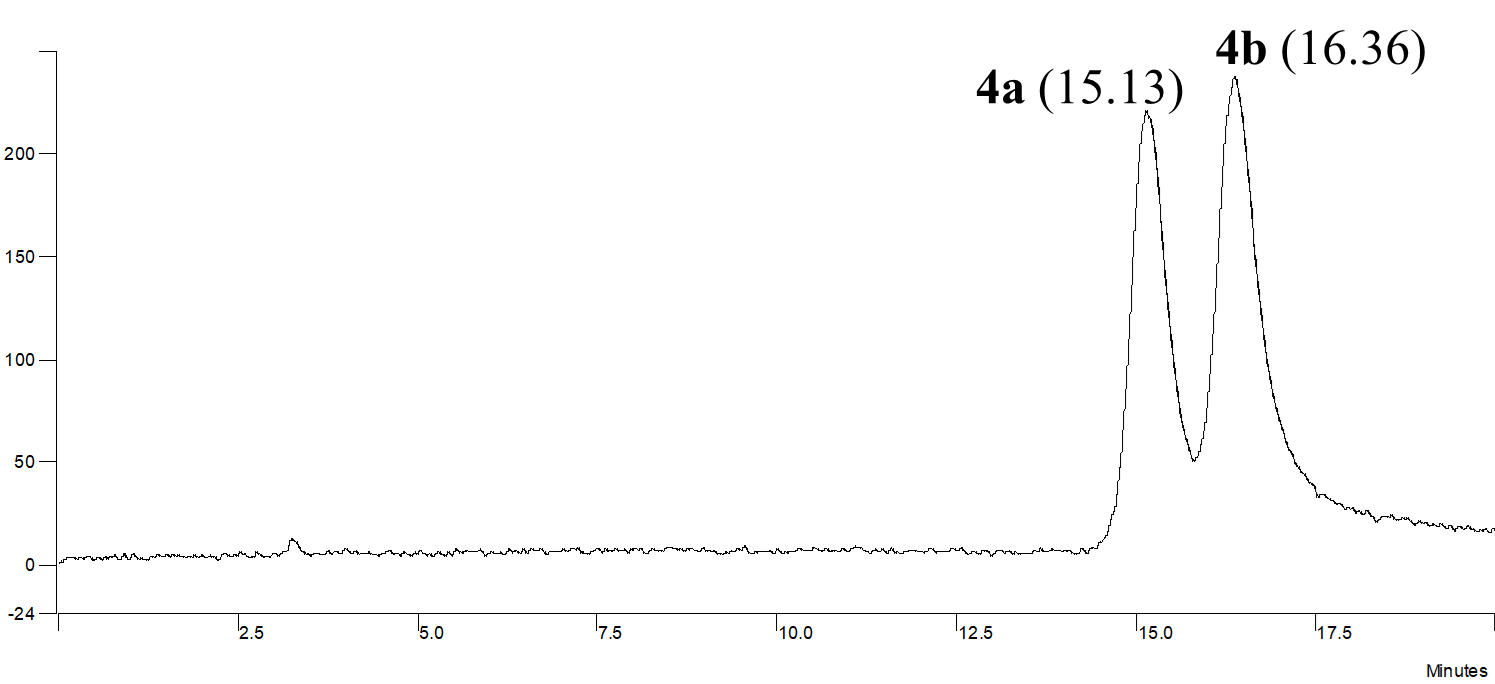

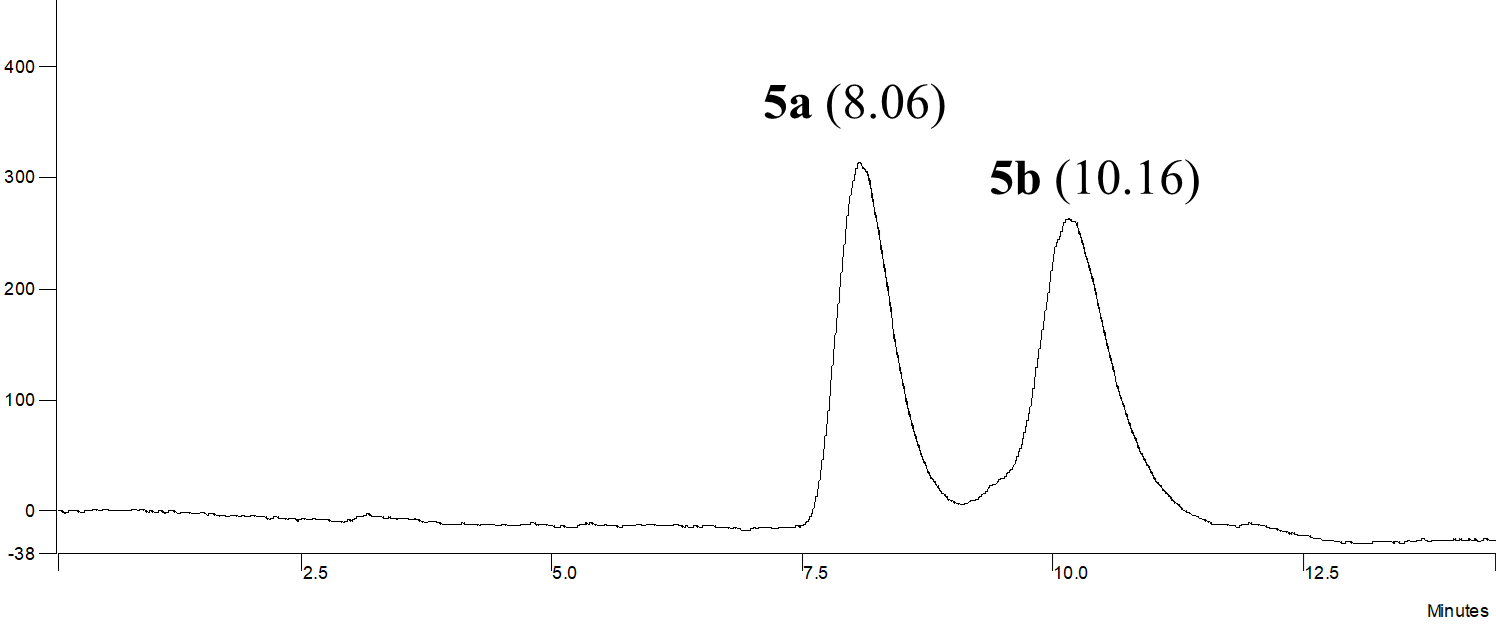

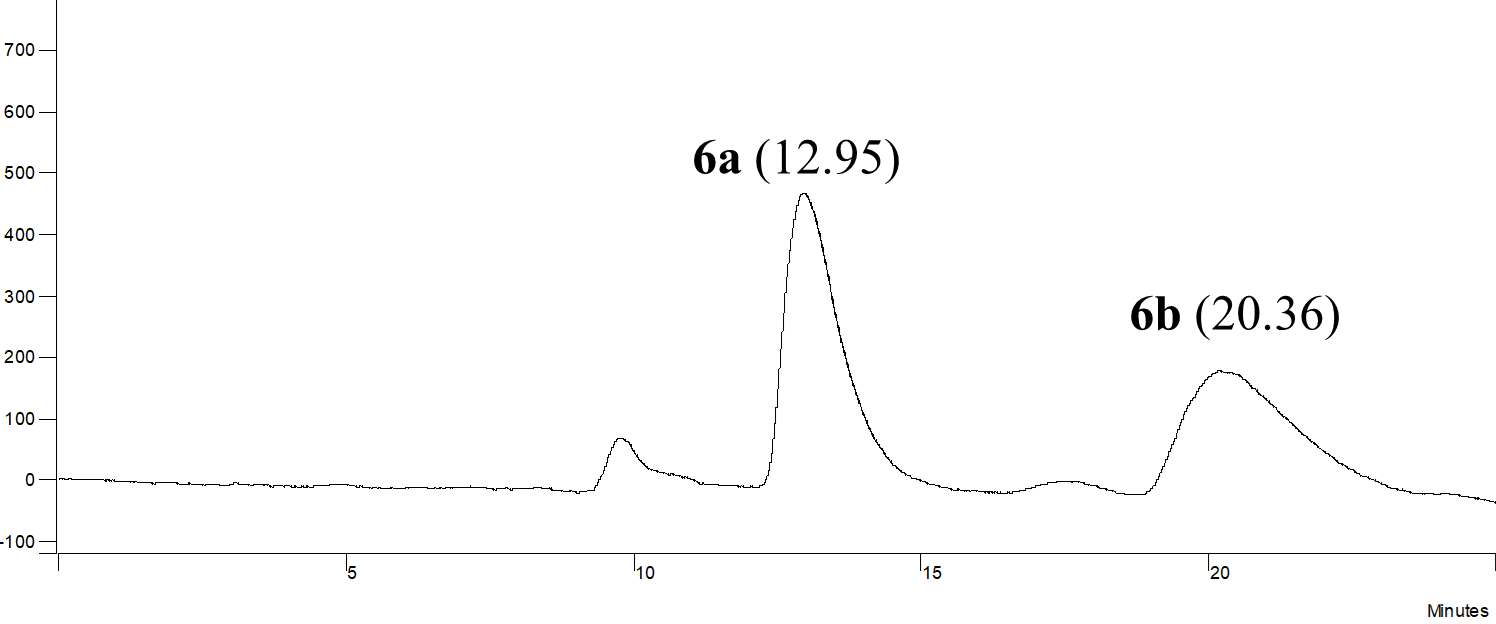

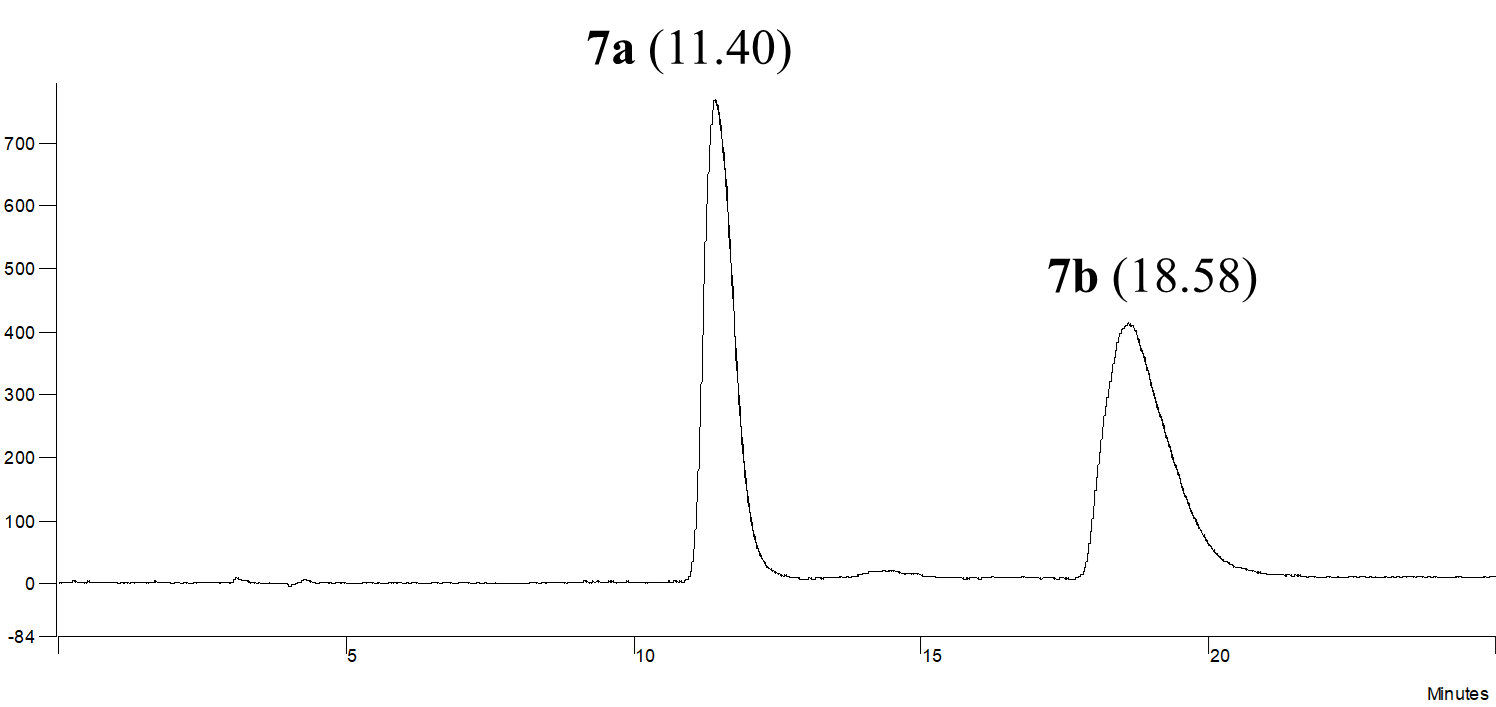


## S.24. Structures of Cudraisoflavone D and Cudraisoflavone I.
